# Colonisation of the colonic mucus gel layer with butyrogenic and hydrogenotropic bacteria in health and ulcerative colitis

**DOI:** 10.1038/s41598-021-86166-6

**Published:** 2021-03-31

**Authors:** Helen Earley, Grainne Lennon, J. Calvin Coffey, Desmond C. Winter, P. Ronan O’Connell

**Affiliations:** 1 School of Medicine and Medical Science, University College Dublin, Belfield, Dublin 4, Ireland; 2 Centre for Colorectal Disease, St Vincent’s University Hospital, Dublin 4, Ireland; 3 Graduate Entry Medical School, Limerick, Ireland

**Keywords:** Microbiome, Bacterial genes, Ulcerative colitis

## Abstract

Butyrate is the primary energy source for colonocytes and is essential for mucosal integrity and repair. Butyrate deficiency as a result of colonic dysbiosis is a putative factor in ulcerative colitis (UC). Commensal microbes are butyrogenic, while others may inhibit butyrate, through hydrogenotropic activity. The aim of this study was to quantify butyrogenic and hydrogenotropic species and determine their relationship with inflammation within the colonic mucus gel layer (MGL). Mucosal brushings were obtained from 20 healthy controls (HC), 20 patients with active colitis (AC) and 14 with quiescent colitis (QUC). Abundance of each species was determined by RT-PCR. Inflammatory scores were available for each patient. Statistical analyses were performed using Mann–Whitney-U and Kruskall-Wallis tests. Butyrogenic *R. hominis* was more abundant in health than UC (*p* < 0.005), prior to normalisation against total bacteria. Hydrogenotropic *B. wadsworthia* was reduced in AC compared to HC and QUC (*p* < 0.005). An inverse correlation existed between inflammation and *R. hominis* (ρ − 0.460, *p* < 0.005) and *B. wadsworthia* (ρ − 0.646, *p* < 0.005). Other hydrogenotropic species did not widely colonise the MGL. These data support a role for butyrogenic bacteria in UC. Butyrate deficiency in UC may be related to reduced microbial production, rather than inhibition by microbial by-products.

## Introduction

The colonic microbiota plays a crucial role in the maintenance of homeostasis in the colon^1^. The short chain fatty acid (SCFA) butyrate is produced by microbial fermentation and is a key metabolite in the gastrointestinal tract^2,3^. It is the primary energy source for colonocytes and also has anti-inflammatory actions, activates mucin release and protects the epithelial barrier^4–8^.

Reduced colonic butyrate concentration has been reported in UC and has been proposed as a potential aetiological factor in the pathogenesis of colonic inflammation^9^. In UC, changes also occur in the composition of the microbiota^10,11^, leading to the hypothesis that microbial factors may contribute to altered butyrate metabolism.

During mucin degradation, by-products are released^12,13^, which may be utilised as substrates for other bacterial species lacking mucolytic properties^13,14^. One such group of hydrogenotropic bacteria utilise these substrates to produce the toxic metabolite hydrogen sulphide (H_2_S)^15–17^, that may induce inflammation through direct toxicity to the epithelium or inhibition of butyrate oxidation^18–20^. The most studied genus is Desulfovibrio^21–23^. However, little information exists on the involvement of other, less abundant species with hydrogenotropic potential in UC (*Desulfobacter, Desulfobulbus* and *Bilophila wadsworthia*)^24,25^. Other commensal species, such as *R. hominis*, are involved in the production of butyrate^26^. Butyrate levels in the colitic colon may be further reduced through reduced production by these species^27–37^.

The outer layer of the MGL provides an energy source and a growth medium for mucus-associated microbiota^38,39^, which represents a community distinct from luminal bacteria^40^. This layer is therefore thought to represent the true host-microbial interface in the human colon^40,41^ . The aims of this study were to determine the relative abundance of butyrogenic (*R. hominis)* and hydrogenotropic (*Desulfobacter, Desulfobulbus* and *Bilophila wadsworthia*) species and their relationship with inflammation within the mucus gel layer (MGL) of the colitic colon. This is the first study to quantitively analyse these species from brushings of the MGL.

## Materials and methods

### Ethical approval, patient recruitment and sample collection

Ethical approval was obtained from the St. Vincent’s University Hospital Ethics and Medical Research Committee. All methods were carried out in accordance with the relevant guidelines and regulations. All volunteers were over 18 years old and written informed consent was obtained. Three patient cohorts were established; healthy controls (HC), patients with quiescent UC (QUC) and patients with active UC (AC). All patient data were anonymised and coded to ensure patient confidentiality.

Healthy controls were recruited when undergoing routine diagnostic day case colonoscopy. No macroscopic evidence of mucosal pathology was evident in these individuals. Patients were excluded from the study if they had a history of antibiotic usage or hospital admission in the six weeks prior to colonoscopy, history of irritable bowel syndrome (IBS), indeterminate colitis, gastrointestinal malignancy or previous colorectal surgery.

Patients with quiescent UC were identified as having previously diagnosed, histologically confirmed UC and were undergoing surveillance colonoscopy. Exclusion criteria were as outlined above or evidence of UC associated dysplasia. Bowel preparations received by all patients undergoing colonoscopy were polyethylene glycol and sodium picosulphate based.

Patients in the AC cohort were recruited prior to undergoing surgical resection for disease refractory to medical management or those with acutely active UC failing to respond to rescue therapy (intravenous steroids, biologics or cyclosporine). Patients had not received bowel preparation prior to undergoing surgery, but had received a single dose of intravenous antibiotics prior to induction of anaesthesia, as per hospital protocol.

A biobank of clinical specimens was present in the form of extracted DNA in the School of Medicine and Medical Sciences in UCD. These DNA extracts were isolated from colonic mucus brushings and stored in sterile micro-centrifuge tubes at − 20 °C. Mucus brush samples were obtained using a Microbiological Protected Specimen Brush (Hobbs Medical Inc., Stafford Springs, Connecticut, USA). Samples were taken from the superficial layer of colonic mucus as previously described^40^. DNA was extracted using a Qiagen DNA Mini Kit (Qiagen, Hilden, Germany). The biobank of samples consisted of DNA from 20 HCs, 14 patients with QUC and 20 patients with AC. For each patient, samples were collected from four areas of the colon; caecum, transverse colon, left colon and rectum.

For each clinical sample the total copy number of bacteria per mg of mucus had previously been determined by quantitative RT-PCR^42^. Inflammatory scores had also been determined as previously described^41^. These data were available in a database for use as part of this study.

### Construction of plasmid DNA standards

A series of plasmid DNA standards was generated to enable calculation of each bacterial copy number in each sample. In brief, freeze dried cultures of reference strains of *Desulfobacter curvatus* (ATCC 43,919)^43^*, Desulfobulbus propionicus* (ATCC 33,891)^43^*, Bilophila wadsworthia* (ATCC49260)^44^ (ATCC, Manassas, V.A., U.S.A.) and a clinical sample of *R. Hominis* were cultured in their respective culture media as previously outlined^42,45,46^. Cultures were placed in a shaking incubator at 200 rpm at 37 °C for 72 h under anaerobic conditions achieved by the use of AnaeroGen anaerobic gas packs (Oxoid, Basingstoke, UK). DNA was extracted using a series of four heat freeze cycles at − 80 °C and 100 °C. Extracted DNA as outlined was used to generate positive controls for each bacterial target. Conventional PCR targeting the 16S rRNA gene of bacterial targets was performed using previously published oligonucleotide primers sourced from Eurofins MWG GmbH (Eurofins MWG GmbH, Ebersberg, Germany) as outlined in Table 1. Assays for *D. propionicus* and *D. curvatus* were carried containing 1.25U GoTaqDNA polymerase (Promega Madison, WI, U.S.A.), 5 × GoTaq Reaction Buffer (Promega), dNTPs at a final concentration of 0.2 mM, forward and reverse primers at final concentrations as outlined in Table 1 and 5 µl of DNA template. Assays for *B. wadsworthia,* and *R. hominis* contained 2X My TaqRed Mix (Bioline, London, UK), primers at concentrations outlined in Table 1. The amplicons generated were cloned into a TOPO vector using the TOPO TA cloning system. DNA from the recombinant plasmid mini-preps were purified using the QIAprep Spin MiniPrep kit (Qiagen). The total weight per recombinant plasmid was calculated and this was used to generate a series of DNA standards of known copy number of the target sequence.

**Table 1:** 16S rRNA targeted PCR primer sequences used in this study and their expected amplicon sizes.

Bacterial target	Forward Primer (5′–3′)	Reverse Primer (5′–3′)	Product Size (bp)	Concentration (nM)	References
*D. propionicus*	CGC GTA GAT AAC CTG TCY TCA TG*	GTA GKA CGT GTG TAG CCC TGG TC	1120	250	^63^
*D. curvatus*	GAT AAT CTG CCT TCA AGC CTG G	CYY YYY GCR RAG TCG STG CCC T*	1150	250	^63^
*B. wadsworthia*	CGT GTG AAT AAT GCG AGG G	TCT CCG GTA CTC AAG CGT G	207	200	^64^
*R. hominis*	TAC TGC ATT GGA AAC TGT CG	CGG CAC CGA AGA GCA AT	230	100	^65^

*Denotes the use of a degenerate base pair. Y = C/T, R = A/G, S = C/G, W = A/T, K = G/T.

### Detection and quantification of microbes in mucus brushings

For each clinical sample the total copy number of bacteria per mg of mucus had previously been determined by quantitative RT-PCR^45^.

RT PCR using an assay specific for the 16S rRNA gene of each bacterial target was performed using primers outlined above. All PCR reactions for the targets *D. propionicus*, *D. curvatus* and *B. wadsworthia* were carried out on an Applied Biosystems7900HT Fast Real-Time PCR machine (Applied Biosystems Foster City, CA, USA.) using the primers outlined in Table 1. Each reaction was performed in duplicate and carried out in an optical grade 384-well plate at a final volume of 20 µl. Each reaction consisted of 1X SyberGreen PCR Master Mix (Applied Biosystems), forward and reverse primers at concentrations outlined in Table 1 and 4 µl of template DNA. For all bacterial targets standard cycling conditions and melt curve analysis were employed, plus additional annealing stages. For *D. propionicus* and *D. curvatus* incubations of 72 °C for 30 s and 79 °C for 10 s were included. For *B. wadsworthia* an additional annealing stage at 79 °C for 10 s was added. Reactions for *R. hominis* were carried out on an Illumina Eco Real Time PCR system machine (Illumina, San Diego, CA, USA) using standard annealing and melt curve analysis. Thermocycling conditions for *R. hominis* were; 40 cycles at 95 °C for 30 s, elongation at 55ºC for 60 s and annealing at 72ºC for 10 s. Each reaction was carried out in an optical grade 48-well plate at a final reaction volume of 10 µl, containing 1X SensiFAST SYBR No-ROX Mix (Bioline) appropriate forward and reverse primers at final concentrations outlined in Table 1 and 4 µl of DNA template. All assays included water as a negative control and positive controls.

### RT-PCR data analysis

Data analysis for all PCR assays performed on the Applied Biosystems platform was performed using SDS 2.4 software (Applied Biosystems). For assays performed on the Illumina platform (Illumina) EcoStudy software v 5.0 (Illumina) was used. Target copy number in each sample was determined based on the fold change (2^-∆Ct^) relative to the 10^7^ copies/µl standard. Copy numbers were normalised for dilution volume, elution volume, DNA concentration and sample weight and for total bacterial copy numbers in each sample.

### Statistical analysis

**Figure 1:**
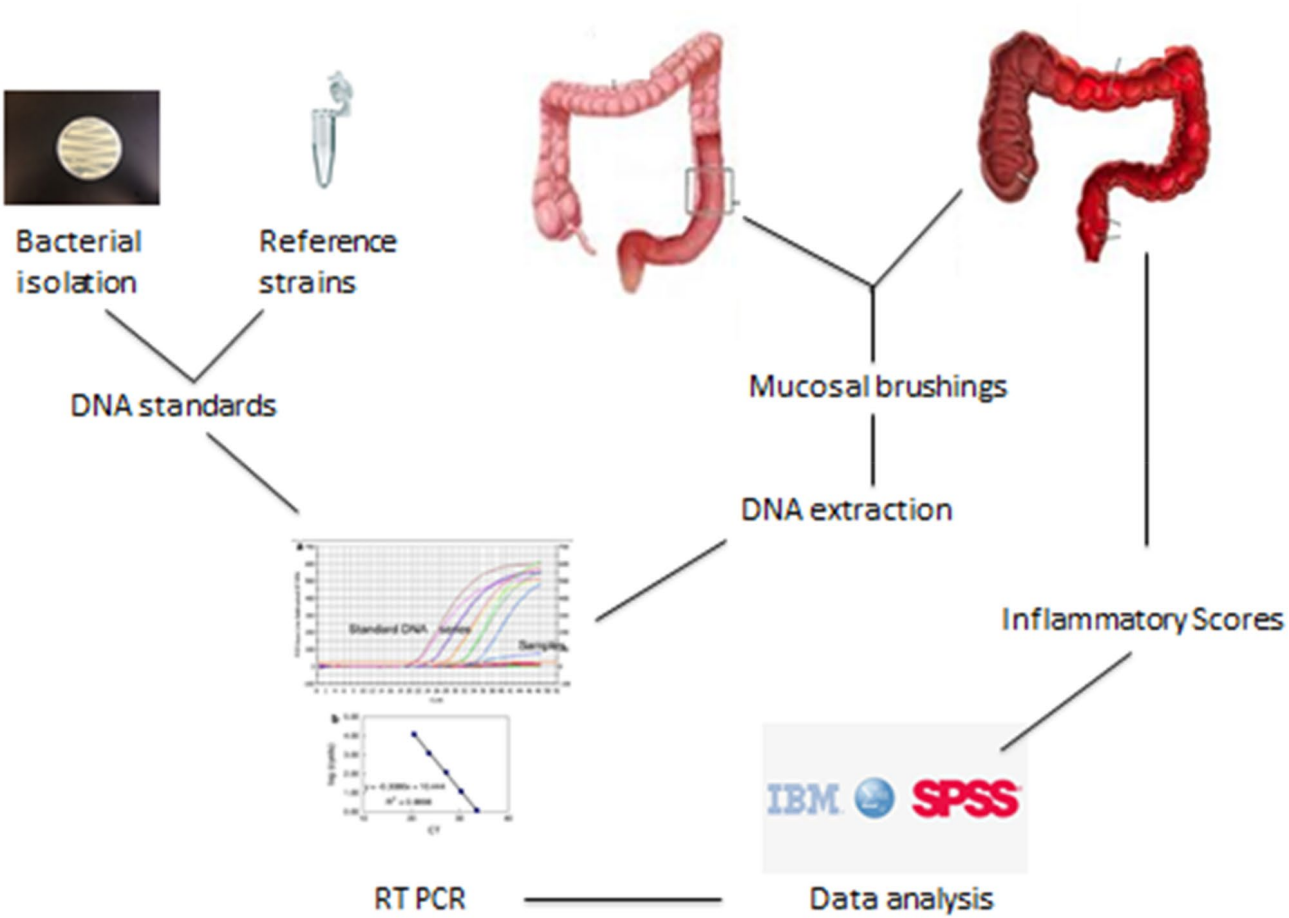
Schematic representing work flow through sample collection to data analysis.

Normalised data were exported to SPSS statistics, version 20.0 (SPSS statistics, IBM, London, U.K.) for statistical analysis. Data were tested for normality of distribution, and statistical comparisons were performed based on Mann–Whitney U test and Kruskal–Wallis comparisons. Methods are summarised in Fig. 1.

## Results

### Patient demographics

Fifty-four individuals were included in the study, comprising 20 patients with AC, 14 with QUC and 20 HC. Patients in the AC group were undergoing colectomy for disease refractory to medical management. No patients in this group had presented with an acute surgical emergency. Of the patients in the quiescent group, 5 underwent colonoscopy to monitor disease activity, the remaining 9 patients were undergoing screening colonoscopy procedures, eight of whom had disease duration of greater than ten years. As a result, the mean time since diagnosis was longer in the quiescent group than in the acute colitis group. In addition, the mean age in the QUC cohort (49 yrs) was also older than in the AC cohort (36 yrs). The mean age in the HC cohort was 48.2 yrs which closely resembled that of the quiescent cohort. The mean Mayo score of AC patients was 10.3, whereas the QUC cohort had a mean of 0.25. Information with regard to smoking status, medication and previous appendectomy was available. A summary of patient demographics is outlined in Table 2.

**Table 2:** Summary of characteristics of individuals in each cohort.

		HC	QUC	AC
n		20	14	20
Age (years)		48.2 (21–77)	50.07 (29–72)	37.4 (23–66)
Gender (M/F)		16/4	10/4	12/8
Time since diagnosis (years)		n/a	18 (10–33)	5 (2–14)
Appendectomy		1	1	3
Smoking Status	Current Former Never	0 4 9	6 2 6	2 3 9
Medication use	Steroid	n/a	1	12
	5-ASA	n/a	7	8
	Azathioprine	n/a	4	0
	Infliximab	n/a	0	12
Mean Mayo Score		0	0.25	10.3

### Colonisation of the colon with butyrogenic species *R. hominis*

**Figure 2:**
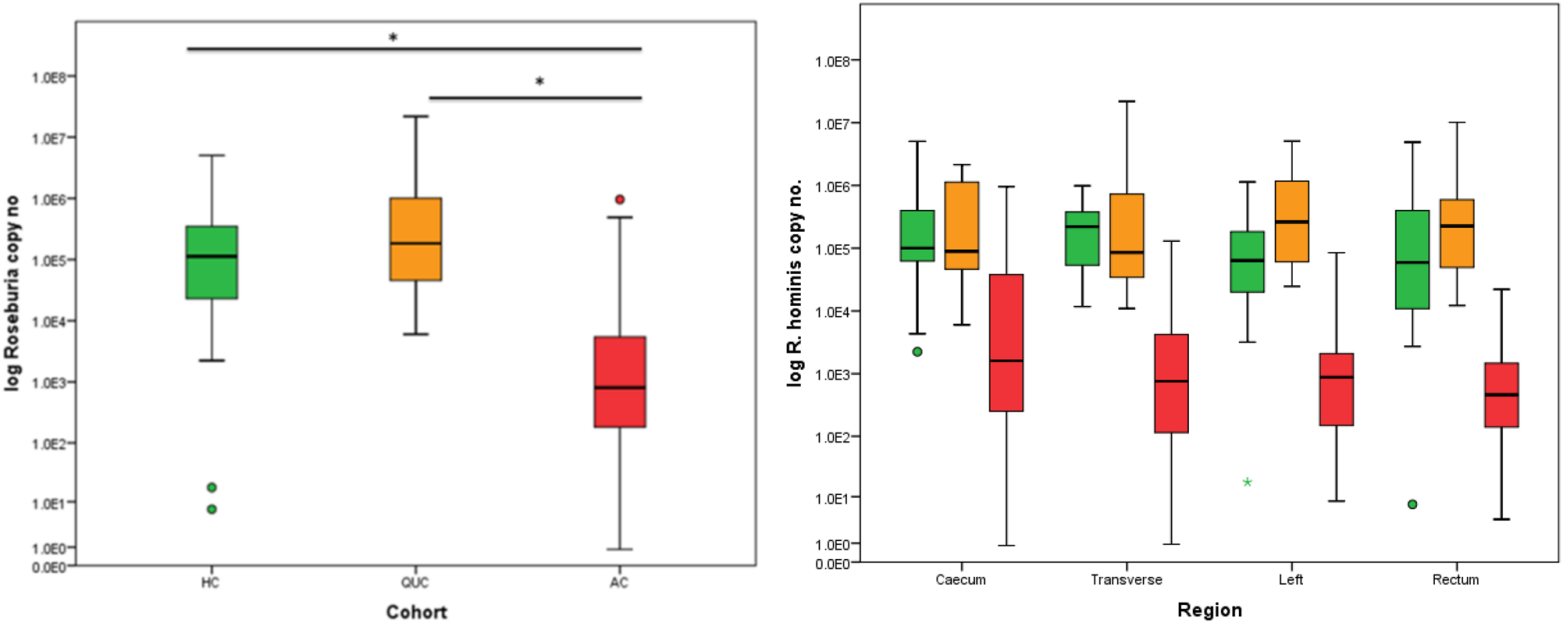
Boxplots representing inter-cohort comparisons of *R. hominis*. Healthy controls are represented in green, patents with quiescent UC in orange and patients with acute UC in red. * denotes *p* < 0.05. Loco regional comparison along the longitudinal axis of the colon in the three patient cohorts.

*R. hominis* was detected in all individuals in the three cohorts. The abundance of *R. hominis* was significantly reduced in AC compared to HC and patients with QUC (Table 3, Fig. 2(i)). No significant difference was detected between the HC and those with QUC (Table 3). After normalisation for total bacterial copy number, no significant difference in relative abundance between any patient cohort was noted (Table 3). The reduction in abundance of *R. hominis* in the acute UC cohort compared to HC and QUC was observed in all four areas of the colon, caecum, transverse colon, left colon and rectum (Table 4). No significant difference in *R. hominis* abundance was detected between health and QUC across all four colonic regions.

**Table 3:** Median copy number/mg and median relative abundance of *R. hominis* after normalisation for total bacterial copy number in each patient cohort.

Cohort	n	Median Copy No	IQR	Cohort Comparison	*p* value
HC	20	1.13E + 5	3.37E + 5	HC-QUC	0.072
QUC	14	1.87E + 5	1.03E + 6	HC-AC	0.000
AC	20	8.06E + 2	5.30E + 3	QUC – AC	0.000

Cohort	n	Median Normalised Copy No	IQR	Cohort Comparison	*p* value
HC	20	3.41E−3	1.72E−2	HC-QUC	0.388
QUC	14	6.89E−3	4.40E−2	HC-AC	0.631
AC	20	5.17E−3	3.31E−2	QUC – AC	0.627

*p* values for inter-cohort comparisons.

**Table 4:** Comparison of *R. hominis* copy number and relative abundance on a loco-regional basis.

R. hominis copy no.												
	n	HC		n	QUC		n	AC		HC-QUC	HC-ACact	QUC-AC
		Median	IQR		Median	IQR		Median	IQR			
Caecum	16	1.00E+5	3.75E+5	11	8.93E+4	1.27E+6	17	1.61E+3	4.56E+4	0.716	0.000	0.001
Transverse	17	2.20E+5	3.51E+5	13	8.56E+4	2.47E+6	16	7.62E+2	4.85E+3	0.869	0.000	0.000
Left	15	6.57E+4	1.85E+5	11	2.62E+5	2.19E+6	16	8.87E+2	2.29E+3	0.039	0.000	0.000
Rectum	15	5.93E+4	5.08E+5	11	2.25E+5	9.55E+5	16	4.64E+2	1.46E+3	0.305	0.000	0.000

Relative abundance of R. hominis												
	n	HC		n	QUC		n	AC		HC-QUC	HC-AC	QUC-AC
		Median	IQR		Median	IQR		Median	IQR			
Caecum	15	1.99E−3	1.33E−2	9	1.04E−2	8.62E−2	13	4.79E−3	1.37E−2	0.387	0.982	0.301
Transverse	16	2.93E−3	2.38E−2	10	8.62E−3	1.27E−2	14	3.37E−3	2.10E−2	0.916	0.934	0.815
Left	15	2.76E−3	2.76E−2	9	1.70E−3	6.84E−2	15	3.70E−3	5.32E−2	0.698	.0.395	0.929
Rectum	15	9.95E−3	1.70E−2	10	7.40E−3	4.45E−2	14	1.02E−2	3.52E−2	1.000	0.727	0.953

### Colonisation of the MGL with hydrogenotropic bacteria

**Figure 3:**
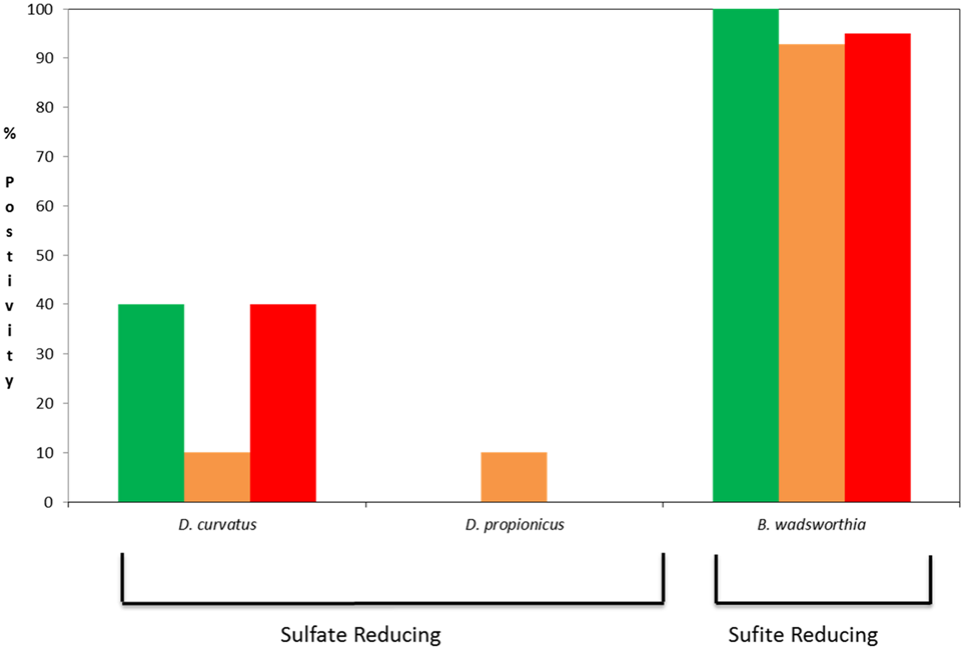
Colonisation rates of each bacterial target in each patient cohort. Healthy controls are represented in green, patients with quiescent UC in orange and patients with acute UC in red.

Low rates of colonisation with the sulphate reducing *D. curvatus* and *D. propionicus* species were detected in all patient cohorts (Fig. 3). 40% of patients were positive for *D. propionicus* in the HC and AC cohorts, and 10% of patients in the QUC cohort. *D curvatus* was detected in 10% of patients with QUC. This species was not detected in healthy individuals or the AC cohort (Fig. 3). Given these findings, these microbes were eliminated from further study.

**Figure 4:**
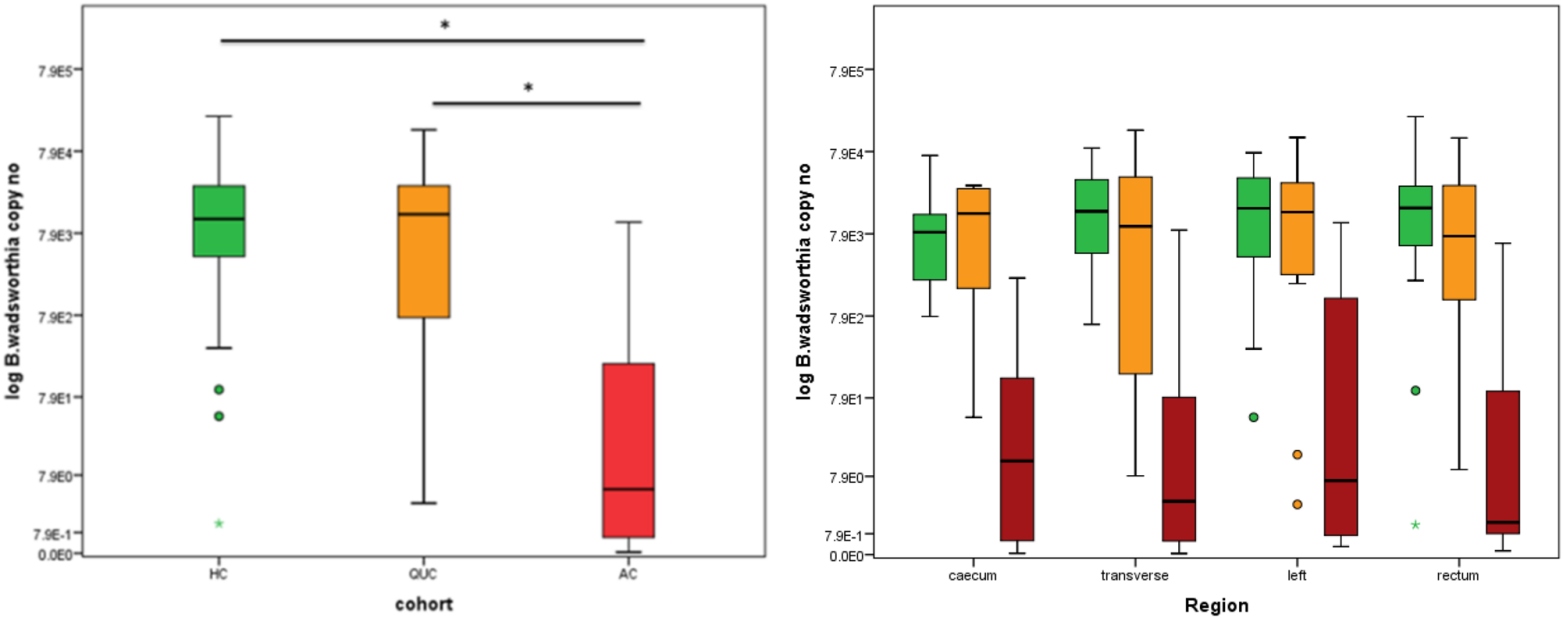
Boxplots representing inter-cohort comparisons of *B. wadsworthia* copy number Healthy controls are represented in green, patients with quiescent UC in orange and patients with acute UC in red. * denotes *p* < 0.05. Comparison between areas of the colon.

*B. wadsworthia* demonstrated a high colonisation rate in all three cohorts (HC 100%, QUC 90%, AC 95%) (Fig. 3). Subsequent quantitative analysis was performed on this species. The abundance of *B. wadsworthia* was significantly reduced in patients with acute UC compared to health and patients with quiescent UC (Table 5, Fig. 4). No significant difference was detected between the healthy controls and those with quiescent UC (Table 5). After normalisation for total bacterial copy number, a persistent significant reduction in the relative abundance of *B. wadsworthia* was noted in the acute UC cohort compared to the healthy cohort (Table 5). No significant difference in normalised *B. wadsworthia* copy number was noted between healthy patients and patients with quiescent UC, or between the quiescent UC group and those with acute colitis (Table 5). The reduction in abundance of *B. wadsworthia* in the acute UC cohort compared to health and quiescent UC was observed in all four areas of the colon, caecum, transverse colon, left colon and rectum. No significant difference in *B. wadsworthia* abundance was detected between health and quiescent UC across all four colonic regions (Table 6, Fig. 4.). After normalisation against total-bacterial copy number, no significant difference was noted between patients with acute UC or quiescent UC compared to health in any of the four colonic regions examined (Table 6).

**Table 5:** Median copy number/mg and median relative abundance of *B. wadsworthia* after normalisation for total bacterial copy number in each patient cohort.

Cohort	n	Median copy No.	IQR	Cohort comparison	*p* value
HC	20	1.18E + 04	2.60E + 04	HC−QUC	0.546
QUC	14	1.35E + 04	3.00E + 04	HC-AC	0.000
AC	20	5.05E + 00	2.32E + 02	QUC–AC	0.000

Cohort	n	Median normalised copy No.	IQR	Cohort comparison	*p* value
HC	20	3.64E–04	1.94E–02	HC-QUC	0.467
QUC	14	7.75E–04	3.27E–03	HC-AC	0.030
AC	20	1.92E–04	1.85E–03	QUC–AC	0.150

**Table 6:** Comparison of *B. wadsworthia* copy number and relative abundance on a loco-regional basis.

*B. wadsworthia* copy no												
	n	HC		n	QUC		n	AC		HC-QUC	HC-AC	QUC-AC
		Median	IQR		Median	IQR		Median	IQR			
Caecum	18	8.39E + 03	1.34E + 04	13	1.40E + 04	2.92E + 04	16	1.02E + 01	1.68E + 02	0.590	0.000	0.000
Transverse	18	1.49E + 04	3.20E + 04	12	9.97E + 03	4.92E + 04	15	5.05E + 00	3.27E + 02	0.611	0.000	0.000
Left	17	1.17E + 04	3.14E + 04	11	1.46E + 04	3.48E + 04	14	7.10E + 00	1.50E + 03	0.869	0.000	0.001
Rectum	19	1.74E + 04	2.86E + 04	13	7.43E + 03	4.14E + 04	13	1.89E + 00	2.02E + 02	0.219	0.000	0.002

Normalised *B. wadsworthia* copy no.												
	n	HC		n	QUC		n	AC		HC-QUC	HC-AC	QUC-AC
		Median	IQR		Median	IQR		Median	IQR			
Caecum	18	2.32E–04	8.63E–03	13	4.44E–04	2.01E–02	16	5.36E–05	1.39E–03	0.701	0.241	0.635
Transverse	18	4.38E–04	1.66E–02	12	9.46E–04	3.02E–03	15	6.47E0–6	2.51E–03	0.421	0.072	0.157
Left	17	2.65E–04	9.10E–03	11	1.34E–03	3.44E–03	14	8.39E–04	1.14E–02	0.729	0.771	0.953
Rectum	19	5.81E–04	6.13E–02	13	4.29E–04	3.88E–03	13	2.55E–04	7.87E–04	0.388	0.189	0.369

### Correlation of butyrogenic and hydrogenotropic bacteria with inflammatory cell infiltrates

To determine whether the abundance of *R. hominis* or *B. wadsworthia* in the colon was associated with markers of inflammation, correlations with inflammatory cell infiltrates were performed. A negative correlation existed between inflammatory cell infiltrate and abundance of *B. wadsworthia,* (ρ − 0.646, *p* = 0.000). A negative association was also found between inflammatory cell infiltrate and abundance of *R. hominis,* (ρ − 0.460, *p* = 0.000).

## Discussion

The present study uses protected specimen brushings of the colonic mucosa as the sampling method^40^. This unique approach to studying the innate microbiota has several advantages. It minimises contamination with luminal contents compared to faecal sampling and obtains a higher proportion of bacterial DNA than host DNA when compared to mucosal biopsies^40,47^. Faecal samples, the MGL and mucosa have each been shown to harbour distinct microbial communities^48^. Mucus brushings are believed to yield findings that are more representative of the true burden of these species within the outer layer of the MGL, which represents the true host-microbial interface in the human colon^39,40,47^.

The primary aim of this study was to perform quantitative analysis of *R. hominis,* a species known to produce substantial amounts of butyrate in vitro^49^. This species widely colonised the MGL in health and UC, indicating that this is an ubiquitous member of the innate microflora. Its’ abundance was reduced in patients with AC compared to health and QUC. This reduction was observed in all four colonic regions, supporting the hypothesis that UC is associated with a global dysbiosis and the concept of spatial homogeneity along the longitudinal axis of the colon^42,48^. An inverse correlation between *R. hominis* abundance and inflammatory cell infiltrates was demonstrated, indicating that depletion of this species is a feature of inflammation in the colitic colon.

In keeping with these data, reductions in the abundance of this species, as well as an inverse correlation with disease severity, have been reported in UC in previous studies based on faecal sampling^28,32^. However, studies of mucosal biopsies yielded conflicting results^50,51^. Willing et al.reported a reduction in *R. hominis* in patients with ileal CD, but not UC^50^, while Lepage et al.reported no reduction in the species in mucosal biopsies of twins with UC^51^. These findings, combined with the observation by Machiels et al.that a stable reduction occurred in the faeces of patients with quiescent disease, led to the hypothesis that alterations in *Roseburia* abundance may play a role in the study of the transient microbiota, rather than the adherent microbiota^28^. The current study challenges this theory, suggesting that the reduction in *R. hominis* contributes to the dysbiosis of the MGL adherent microbiota in the inflamed colon, with abundances returning towards healthy levels when disease is in remission. *R. hominis* has been shown to have anti-inflammatory mechanisms in vitro^52^. It is tempting to hypothesise that this is related to the production of butyrate however, the authors acknowledge that this cannot be inferred from compositional data alone.

This study also aimed to determine colonisation patterns of H_2_S producing species. *Desulfovibrio* remains the most studied of these bacteria and to date a consensus has not been reached as to their precise role in UC^25,45,53,54^. Few studies analysed other low-abundance microbes such as *D. desulfuricans* and *D. propionicus,* both of which are capable of foraging sulfate and producing H_2_S^24^. No data currently exist pertaining to the involvement of these species in UC. This study demonstrated low colonisation rates of *D. desulfuricans* and *D. propionicus* in both health and UC. 40% of healthy subjects were positive for *D. curvatus* in the current study, in keeping with data from Gibson et al*.,* who reported hydrogenotropic species in 50% of faecal samples^55^. On the other hand, it is at odds Nava et al.who detected both species in mucosal biopsies of 25 healthy subjects^56^. It should also be noted that in the aforementioned study, the species *Desulfovibrio* accounted for the majority of hydrogenotropic present. Previous work from our laboratory demonstrated a high rate of colonisation with *Desulfovibrio*^45^ in these patient samples. *Desulfovibrio* may be the dominant species of sulphate reducing bacteria colonising this patient cohort also. Given the low colonisation rates of *D. desulfuricans* and *D. proprionicus* in UC, there is no compelling evidence of an association between these species and UC in the present study.

*B. wadsworthia* was present at detectable levels in the majority of individuals in this study. These data demonstrate a significant reduction in *B. wadsworthia* in patients with AC compared to healthy individuals and those with QUC. The reduced abundance in patients with AC compared to healthy individuals remained significant after normalisation against total bacterial counts. The inverse correlation between *B. wadsworthia* abundance and inflammatory score confirm that a reduction in abundance of this species is a feature of the inflamed colon. Although these findings are not in keeping with the hypothesis of increased activity of hydrogenotropic bacteria in UC, the finding is nonetheless significant. These data suggest a tentative link between this species and dysbiosis in AC.

These results are consistent with previously published findings using faecal samples, reporting a reduction in *B.wadsworthia* in patients with Crohn’s disease before treatment compared to health^25^. However it should be noted that these findings differ from those in murine models, where *B. wadsworthia* has been reported to act as a pathobiont, promoting the development of inflammation^57,58^. Evidence suggests that In vivo results of murine models cannot always be extrapolated easily to humans^59,60^, which should be considered when interpreting the contrasting results pertaining to this species reported in human and murine models.

In this study, normalisation of data against total bacterial copy number was performed to reduce potential reporting errors by minimising the effect of between-sample variation and taking the efficiency of the quantification procedure into account for both species investigated. These data are more representative of the actual burden of the target in the MGL. However, normalisation negated the significant difference in abundance observed between health and acute UC when analysis was based on raw copy numbers at each of the four colonic regions examined. This may be due to the fact that individually, these species account for a small proportion of the overall bacterial load at any given site in the colon.

It is likely that several factors contribute to the observed reduction in abundance in these species in UC. Changes in the ecosystem of the MGL as a result of the inflammatory process may result in suboptimal conditions for bacterial survival. To date, no study has specifically addressed the potential interactions between hydrogenotropic, butyrogenic and other species within the MGL in the colitic colon. The authors have previously demonstrated a reduction in mucolytic bacteria in UC^41^, which due to reduced mucin degradation, may lead to a lack of substrate for these bacteria. Hydrogenotropic bacteria have also been shown to impact host physiology^61^. It is possible that *B. wadsworthia* has properties that enable SCFA production. A recently published study demonstrated an association between *B. wadsworthia* and higher butyric acid concentrations in faecal samples of healthy infants^62^, suggesting a potential link between the species and SCFA production in the human colon.

This study investigated the microbiological basis of UC as an energy deficiency disease, with specific focus on butyrogenic and hydrogenotropic bacteria at the level of the MGL. In keeping with the hypothesis that UC is associated with reduced butyrate concentrations, reductions in the abundance of *R. hominis* were noted. However reduced abundance of the hydrogenotropic *B. wadsworthia* was also observed in the colitic colon in this study. These data suggest that the reported lack of butyrate in the colitic colon may be a result of a lack of butyrogenic bacteria, rather than bacterial inhibition by microbial by-products such as H_2_S. Furthermore, altered abundances of these species are associated with active inflammation in UC. Overall, these data suggest that reductions in these species are a feature of the altered microbial signature of the MGL in UC.

## Limitations

The authors acknowledge that the current study has some limitations. It is possible that the use of bowel preparation may have resulted in a loss of some loosely adherent microbes in the patients undergoing colonoscopy (HC ad), however, this would not account for the low colonisation rates in patients with acute UC, as this cohort did not receive bowel preparation prior to surgery. This would be difficult to control for, as adequate bowel preparation is a prerequisite for successful colonoscopy. This study did not determine whether the observed alterations in microbes equated to alterations in butyrate levels in the colon. Further functional studies are warranted to validate these findings. Finally, as with many studies of the microbiota, the authors acknowledge that it is not possible to determine from these data whether the observed changes are a potential driving force behind the pathogenesis of inflammation, or arising as a result of the inflammatory process. The association between these microbial changes and UC warrants further investigation, with a view to establishing whether a cause-effect relationship indeed exists, and whether manipulation of the dysbiosis in UC will have clinical applications in the future.
